# Type 1 interferon suppresses expression and glucocorticoid induction of glucocorticoid-induced leucine zipper (GILZ)

**DOI:** 10.3389/fimmu.2022.1034880

**Published:** 2022-11-23

**Authors:** Wendy Dankers, Melissa Northcott, Taylah Bennett, Akshay D’Cruz, Rochelle Sherlock, Linden J. Gearing, Paul Hertzog, Brendan Russ, Iolanda Miceli, Sebastian Scheer, Maki Fujishiro, Kunihiro Hayakawa, Keigo Ikeda, Eric F. Morand, Sarah A. Jones

**Affiliations:** ^1^ Centre for Inflammatory Diseases, Monash University, Melbourne, VIC, Australia; ^2^ Centre for Innate Immunity and Infectious Diseases, Hudson Institute of Medical Research, Melbourne, VIC, Australia; ^3^ Institutes for Environmental and Gender Specific Medicine, Juntendo University Graduate School of Medicine, Chiba, Japan; ^4^ Department of Internal Medicine and Rheumatology, Juntendo University Urayasu Hospital, Chiba, Japan

**Keywords:** interferon, GILZ, glucocorticoid, STAT1, systemic lupus erythematosus (SLE), inflammation, autoimmunity

## Abstract

SLE is a systemic multi-organ autoimmune condition associated with reduced life expectancy and quality of life. Glucocorticoids (GC) are heavily relied on for SLE treatment but are associated with detrimental metabolic effects. Type 1 interferons (IFN) are central to SLE pathogenesis and may confer GC insensitivity. Glucocorticoid-induced leucine zipper (GILZ) mediates many effects of GC relevant to SLE pathogenesis, but the effect of IFN on GC regulation of GILZ is unknown. We performed *in vitro* experiments using human PBMC to examine the effect of IFN on GILZ expression. JAK inhibitors tofacitinib and tosylate salt were used *in vivo* and *in vitro* respectively to investigate JAK-STAT pathway dependence of our observations. ChiP was performed to examine glucocorticoid receptor (GR) binding at the GILZ locus. Several public data sets were mined for correlating clinical data. High IFN was associated with suppressed GILZ and reduced GILZ relevant to GC exposure in a large SLE population. IFN directly reduced GILZ expression and suppressed the induction of GILZ by GC *in vitro* in human leukocytes. IFN actions on GILZ expression were dependent on the JAK1/Tyk2 pathway, as evidenced by loss of the inhibitory effect of IFN on GILZ in the presence of JAK inhibitors. Activation of this pathway led to reduced GR binding in key regulatory regions of the GILZ locus. IFN directly suppresses GILZ expression and GILZ upregulation by GC, indicating a potential mechanism for IFN-induced GC resistance. This work has important implications for the ongoing development of targeted GC-sparing therapeutics in SLE.

## 1 Introduction

Systemic lupus erythematosus (SLE) is a systemic autoimmune condition predominantly affecting young women. It is a clinically heterogeneous disease with the potential to affect any organ system, and ranges from mild rashes and arthritis through to severe, life threatening renal and central nervous system disease (1). Few treatment advances in SLE have occurred in recent decades, which has resulted in a heavy reliance on glucocorticoids (GC) for the management of SLE. Whilst utilized for their immunosuppressive and anti-inflammatory properties, GC are associated with a myriad of detrimental metabolic effects (2). High doses of GC are often required to gain disease control in severe SLE, potentiating adverse effects and indicating a degree of GC resistance.

Whilst the pathophysiology of SLE remains incompletely defined, a pivotal role for the type 1 interferon (IFN) family has emerged, as evidenced by the overexpression of interferon-stimulated genes (ISG) in 60-80% of SLE patients (3, 4), and more recently by the success of an IFN receptor (IFNAR) blocking agent in improving clinical disease activity in clinical trials (5). The IFN pathway is most likely activated in SLE patients by immune complexes containing host nucleic acids (6). These stimulate Toll-like receptors in plasmacytoid dendritic cells (pDCs) to produce IFN, which in turn interacts with IFNAR, activating janus kinase 1 (JAK1) and tyrosine kinase 2 (Tyk2). This subsequently leads to the phosphorylation of signal transducer and activator of transcription (STAT) proteins. STAT1, STAT2 and interferon-regulatory factor 9 (IRF9) complex to form interferon-stimulated gene factor 3 (ISGF-3) which binds to interferon-stimulated response elements (ISRE), leading the transcription of ISG (7). Serum IFN has been difficult to measure in SLE patients, and consequently ISG have become a widely accepted surrogate measurement (5).

Despite their widespread use, GC have been shown to have poor ability to suppress ISG in patients with SLE except at very high doses, raising the possibility that IFN may be involved in inducing a GC resistant state (8). Recently, we have shown that IFN inhibits the expression of many GC induced genes, further implicating IFN in GC resistance in SLE (9).

GC, in conjunction with the glucocorticoid receptor (GR), acts as a transcription factor, with the ability to both induce and repress gene transcription (10). Glucocorticoid induced leucine zipper (GILZ) is an anti-inflammatory protein which is potently induced by the ligand-bound GR (11). GILZ has been shown to have a multitude of immunosuppressive effects relevant to SLE pathogenesis, including B lymphocyte regulation (12), and GILZ-deficiency exacerbates a murine model of SLE (13). The effect of IFN on GILZ is previously unreported.

Here we provide evidence that IFN suppresses GILZ. We show that GILZ is inversely correlated with ISGs in SLE patients and that GILZ is rapidly down regulated on exposure to IFN in human leukocytes. We demonstrate that transcriptional inhibition of GILZ by IFN signaling is mediated by Jak1/Tyk2, and importantly, show that IFN impairs GC induction of GILZ by reducing GR binding at the GILZ locus. This work identifies a novel mechanism *via* which IFN may induce GC resistance in interferonopathies such as SLE.

## 2 Methods

### 2.1 PBMC isolation and cell culture

Whole blood samples were collected from consenting healthy volunteers. PBMC were isolated using SepMate tubes (Stemcell Technologies) and Histopaque density medium (Sigma-Aldrich). Cells were cultured at 1-2 million cells/mL in RPMI (Gibco) supplemented with 10% autologous serum and Penicillin-Streptomycin-Glutamine (Gibco). Cells were treated with 1000 IU/mL recombinant human interferon alpha 2a (IFN-α; Sigma-Aldrich) for 3 hours unless otherwise indicated. Where applicable, 100 nM dexamethasone (DEX) (Sigma-Aldrich) was added after 1 hour of culture. For experiments including the Jak1/Tyk2 inhibitor tosylate salt (TS, Sigma-Aldrich), cells were treated with 0.25 or 1 µM TS for 1 hour prior to IFN-α addition. Studies were approved by the Monash Health human research ethics committee.

### 2.2 RT-PCR

RNA was extracted using TriReagent (Life Technologies) or the PureLink RNA Mini Kit (Life Technologies) and cDNA was prepared using the High-Capacity cDNA Reverse Transcription Kit (Applied Biosystems) following manufacturer’s instructions. RT-PCR was performed using TaqMan Gene Expression Mastermix (Applied Biosystems) and expression assays (all ThermoFisher) for GILZ (assay ID Hs00608272_m1) and RSAD2 (assay ID Hs00369813_m1). Expression was normalized to 18s rRNA using the ∆∆Ct method (14) with 18s RNA as an endogenous control. Data was acquired on a QuantStudio 6 and analyzed with QuantStudio Real-Time PCR Software.

### 2.3 Flow cytometry

Cultured PBMC were stained with Fixable Viability Dye eFluor506 (eBioscience) and subsequently fixed with 2% paraformaldehyde. After permeabilization (Intracellular Staining Permeabilization Wash Buffer, BioLegend), cells were stained intracellularly for GILZ (clone CFMKG15, eBioscience) or the corresponding rat IgG2a kappa isotype control (clone eBR2a, eBioscience). Data was acquired on the FACSCantoII (BD Biosciences) and analyzed using FlowJo v10.8 (BD).

### 2.4 Public data set analysis

#### 2.4.1 Data set GSE 88884

Baseline microarray data from 1,756 adult SLE patients enrolled in the ILLUMINATE-1 and ILLUMINATE-2 clinical trials (GSE88884), as well as microarray data from 60 healthy controls was downloaded. Data had been quartile normalized and log2 transformed (15). Clinical data, including prednisolone dose, SLE disease activity index (SLEDAI), complement levels and anti-dsDNA levels were kindly provided by Eli Lily. Patients were stratified into IFN over-expressing (IFN high) and normal IFN (IFN low) groups using a validated four gene ISG signature (*IFI27, IFI44, ILI44L, RSAD2*) (16). An IFN score was calculated by normalized the expression of each of the 4 genes using the mean and standard deviation (SD) of the control group, then averaging these normalized values, such that a patients IFN score represents the number of standard deviations their ISG expression is above or below the healthy control mean expression level. Patients with a combined normalized gene expression level of more than two SD from the control mean classified as IFN high. In cases where more than one probe represented a single gene, normalized probe readings were averaged.

#### 2.4.2 Other datasets

Microarray data from GSE123549, GSE138064 and GSE16251 was downloaded, and GILZ expression analyzed by averaging all expressed GILZ probes within each dataset. ChIP-seq data from GSE99887, GSE107584 and GSE43036 was directly loaded into the UCSC Genome Browser (human genome version Hg19) using the ‘custom tracks’ functionality.

### 2.5 Tofacitinib treated mice

cDNA extracted from the kidneys of NZB/NZW F1 mice treated with or without tofacitinib and dexamethasone was analyzed for the expression of GILZ as previously described (17). GILZ expression was then normalized using the ∆∆Ct method (14) with β-actin as the endogenous control. Primers used were: β-actin forward (5’-CATCCGTAAAGACCTCTATGCCAAC-3’), β-actin reverse (5’-ATGGAGCCACCGATCCACA-3’), GILZ forward (5’- CAGCAGCCACTCAAACCAGC-3’) and GILZ reverse (5’- ACCACATCCCCTCCAAGCAG-3’).

### 2.6 Chromatin immunoprecipitation

Samples were prepared for ChIP using the TruChIP Chromatin Shearing Kit (Covaris) following the High Cell Protocol. For each condition 20 million PBMC per condition were fixed in 1% formaldehyde for 7.5 minutes and frozen as dry cell pellets. The next day, nuclei were isolated and chromatin sheared in the Covaris S220 sonicator for 15 minutes. After sonication ChIP was performed overnight at 4°C in Covaris IP Dilution Buffer with 20 uL protein A magnetic beads while rotating. Half of each sample was ChIPped with 5 µg anti-GR antibody (clone G-5, Santa Cruz BioTechnologies) and the other half with 5 µg mouse IgG2b isotype control antibody (Cell Signaling Technology). Beads were then washed with low salt buffer (50 mM HEPES pH 7.5, 150 mM NaCl, 1 mM EDTA, 1% Triton X-100, 0.1% sodium deoxycholate), high salt buffer (50 mM HEPES pH 7.5, 500 mM NaCl, 1 mM EDTA, 1% Triton X-100, 0.1% sodium deoxycholate), lithium chloride buffer (10 mM Tris-HCl pH 8.0, 1 mM EDTA, 0.5% sodium deoxycholate, 0.5% NP-40, 250 mM LiCl) and then TE buffer (10 mM Tris-HCl pH 8.0, 1 mM EDTA, pH 8.0). DNA was then eluted in elution buffer (1% SDS, 100mM NaHCO3). Crosslinks were reversed by adding NaCl to a final concentration of 0.2 M and incubation overnight at 66°C. Proteins were digested by protease K, and DNA purified with phenol/chloroform extraction follow by ethanol precipitation. Enrichment of GR binding at GILZ loci was determined with RT-PCR using the SYBR Green Mastermix (Life Technologies) and primers shown in **Table 1**, with enrichment calculated as % of the total input control.

**Table 1:** Primer sequences for ChIP-qPCR studies of the GILZ locus.

Region	Forward	Reverse
A	CTGCAGAACGAACCCAAAGC	TTCCCTTCAACTCCAGCTGG
Z	GAAGGAGCAAGAGGGGCAG	ACTGATTCATGGGTACTGGCC
K	TTGGAAGCCGCCTAAGAACC	TTTCTCCTCCCCGTCCTCC
L	GCACTTGGAACCACAAACCC	GGTGCAGGGCTCAAACAATG

### 2.7 Western blot

Cells were lysed in RIPA buffer (150 mM NaCl, 1% IgePal, 5 g/L sodium deoxycholate, 0.1% SDS, 50 mM Tris-HCl pH 8.0, 1x protease inhibitor, 1x phosphatase inhibitor) and protein amount quantified with the BCA Protein Assay Kit (Pierce). 27.5 µg protein was run on a NuPage 4-12% Bis-Tris protein gel (Invitrogen) in Laemmli buffer and 1x NuPage Sample Reducing Agent (Invitrogen), and subsequently blotted onto a PVDF membrane. Membranes were stained for β-actin (Sigma-Aldrich), phosphorylated STAT1 (Thermo Fisher), or total STAT1 (Thermo Fisher) overnight at 4°C in TBS-T, followed by secondary staining with goat-anti-mouse-HRP antibody (Cayman Chemical). Western blots were analysed with ECL Western blotting reagents (GE Healthcare) on the ImageQuant LAS 4000 (GE Healthcare).

### 2.8 Patient and public involvement

Patients and members of the public were not involved in the design of these studies.

### 2.9 Statistical analysis

Data was tested for normality using the Shapiro-Wilk test. Normally distributed data was then analysed for statistically significant differences using the Student’s T-test or ANOVA with Tukey’s post-test for two or more conditions, respectively. Non-normally distributed data was tested using the Mann-Whitney test for two conditions. For comparing to a normalized control value of 1, a one-sample t-test was used. Correlation was assessed using Spearman’s correlation. Results were considered statistically significant if p<0.05. Data was analysed using GraphPad Prism v9.1.

## 3 Results

**Figure 1:**
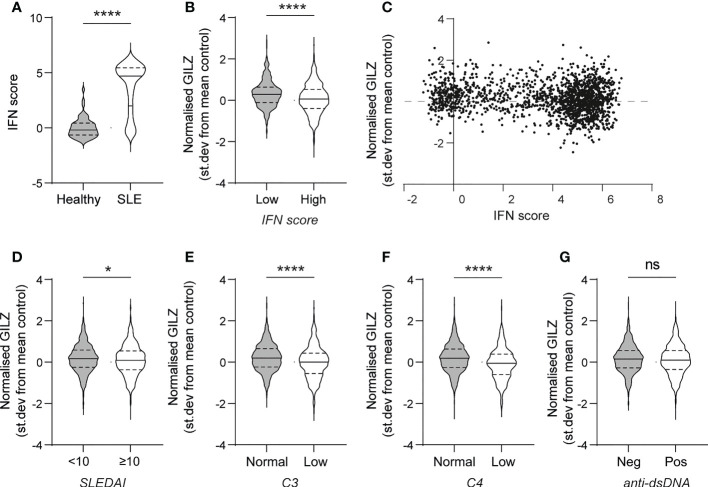
PBMC from SLE patients with high IFN score and higher disease activity express less GILZ. **(A)** IFN score in PBMC of healthy controls and SLE patients. **(B)** GILZ expression in PBMC of SLE patients with low (n=439) or high IFN (n=1317)score. **(C)** Correlation between IFN score (expressed as STD from mean healthy control expression level) and GILZ mRNA expression. **(D–G)** GILZ expression in PBMC of SLE patients, categorized by SLEDAI **(D)**, C3 and C4 complement activity **(E–F)** and anti-dsDNA status **(G)**. All data originated from GSE88884 (n=1,756 SLE patients, n=50 healthy controls). *p<0.05, ****p<0.0001. ns, Not significant.

First, we analysed the relationship between GILZ mRNA expression and ISGs in a large cohort of SLE patients (GSE88884, n=1,796). As expected, SLE patients had over-expression of ISGs compared to healthy control subjects (median [IQR] normalized expression level -0.19 [-0.65- 0.43] vs 4.70 [2.00-5.44], p<0.0001) (**Figure 1A**). GILZ mRNA expression was lower in IFN-high SLE patients (n=1317) compared to IFN-low SLE patients (n=439) (0.10 [-0.36 - 0.56] vs 0.26[-0.12 – 0.61], p=0.0003) (**Figure 1B**), despite higher GC use in the IFN high group (IFN high median [range] prednisolone dose 10 (0–125)mg/day vs IFN low 5 (0–40)mg/day, p<0.001, data not shown). There was a weak negative correlation between IFN score and GILZ expression in this cohort (**Figure 1C**). Patients with high disease activity (SLEDAI score >/=10) had modest suppression of GILZ expression compared to patients with lower disease activity (SLEDAI<10) (**Figure 1D**). Similarly, patients with low complement levels had lower GILZ expression (**Figures 1E, F**). We previously published mouse model data showing GILZ deletion exacerbates clinical severity of disease without affecting autoantibody production (13), and likewise here we observed no difference in GILZ expression between patients with and without anti-dsDNA antibodies (**Figure 1G**).

**Figure 2:**
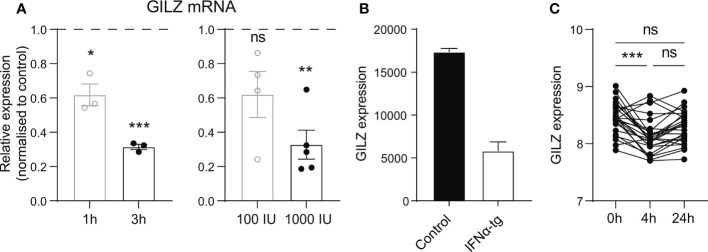
Type I IFN inhibits GILZ expression in human and mouse. **(A)** Healthy PBMC were treated with 1000 IU IFNα for 1 or 3 hours (left panel), or with 100 IU or 1000 IU for 3 hours (right panel), after which GILZ expression was analysed by qPCR. n=3-5, pooled from at least three independent experiments. **(B)** GILZ expression in splenocytes from WT or IFNα-overexpressing mice (GSE123549, n=2). **(C)** GILZ expression in PBMC from MS patients, before and 4 or 24 hours after injection of 500 µg IFNβ (GSE138064, n=25-26). *p<0.05, **p<0.01, ***p<0.001. ns, Not significant.

To verify whether these correlations were directly linked to type I IFN, we tested the effect of IFN on GILZ expression in *ex vivo* healthy human PBMC. We found that recombinant human IFNα reduced GILZ expression in a time- and dose-dependent manner (**Figure 2A**). We also analysed the effects of IFN on GILZ expression in a variety of conditions from several publicly available datasets. Splenocytes from IFNα transgenic mice (GSE123549) had lower GILZ expression than control cells (**Figure 2B**). Patients with multiple sclerosis treated with therapeutic recombinant interferon-β had suppressed GILZ expression compared to patients who did not receive this treatment (GSE138064) (**Figure 2C**).

**Figure 3:**
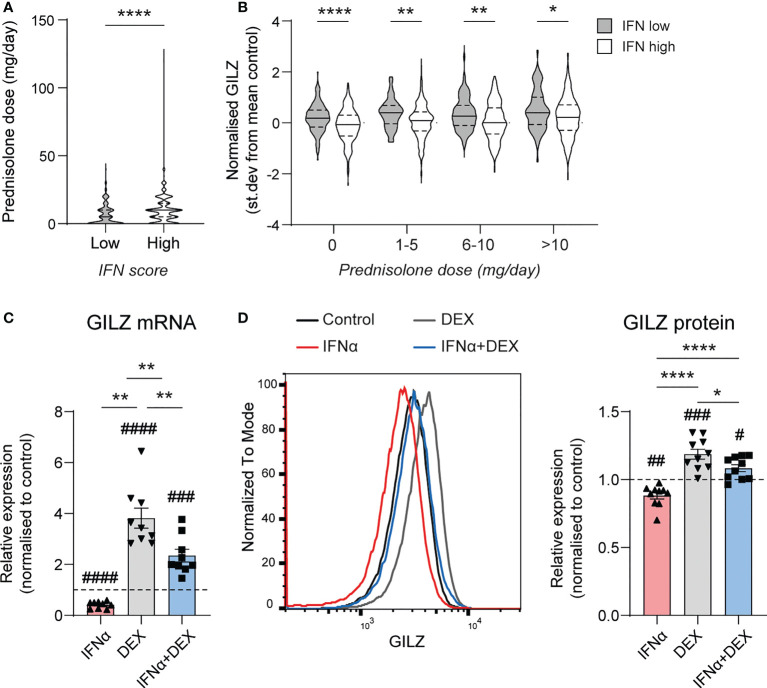
Type I IFN inhibits DEX-induced GILZ. **(A)** Daily prednisolone dose in SLE patients with low or high IFN score (GSE88884, n=1756). **(B)** GILZ expression in PBMC of SLE patients stratified by daily prednisolone dose (GSE88884, n=1756). **(C, D)** Healthy PBMC were treated with IFNα, DEX or both, after which GILZ mRNA expression was analysed by qPCR **(C)**, and GILZ protein level was analysed by flow cytometry in the same samples **(D)** GILZ protein level is represented as MFI normalized against the control MFI for each donor. n=9, pooled from three independent experiments. *p<0.05, **p<0.01, ****p<0.0001. #symbol indicates significance of difference from control (untreated) cells, # p<0.05, ## p<0.01, ### p <0.001, ####p<0.0001.

Since GILZ is an important target for the immunosuppressive actions of GC, we next analysed the effect of IFN on GC-induced GILZ induction in SLE patients from dataset GSE88884. Overall, we noted that IFN-high patients receive more GC (median [IQR] dose 9.5[2.5-15]mg vs 5.0 (0–10)mg, p<0.0001)(**Figure 3A**), but had lower GILZ expression as discussed above (**Figure 1B**), suggesting impaired GC-induced GILZ expression in IFN-high patients. When matched for GC dose, IFN-high patients had lower GILZ expression that IFN-low patients (**Figure 3B**). We verified this effect *ex vivo* in human PBMC, showing that IFNα reduced GC induction of GILZ at both the mRNA and protein level (**Figures 3C, D**).

**Figure 4:**
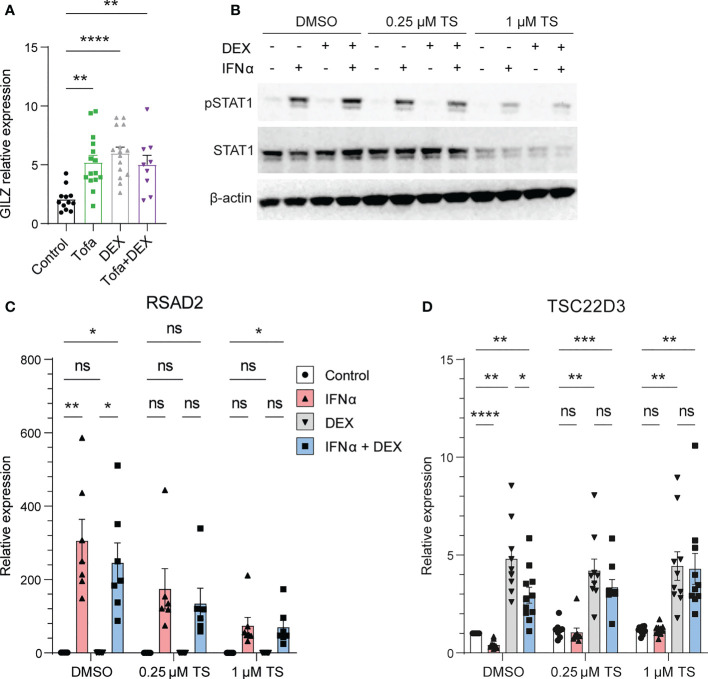
JAK1/Tyk2 signaling is involved in IFN-mediated inhibition of GILZ. **(A)** Kidneys from lupus-prone BWF1 mice treated with tofacitinib, DEX or both were analysed for GILZ expression by qPCR (n=9-15). **(B–D)** Healthy human PBMC were treated with the Jak1/Tyk2 inhibitor tosylate salt (TS) before exposure to IFNα, DEX or both. Expression of pSTAT1 and STAT1 was analysed by western blot **(B)**, and RSAD2 **(C)** and GILZ **(D)** by qPCR. n=9-11, pooled from three independent experiments. *p<0.05, **p<0.01, ***p<0.001, ****p<0.0001. ns, Not significant.

We next investigated the mechanisms behind IFN regulation of GILZ. In mice treated with tofacitinib, a pan JAK inhibitor, GILZ expression was elevated to a level comparable to dexamethasone (Dex) treatment. The effect of tofacitinib and Dex was not additive. (**Figure 4A**). Moreover, when healthy human PBMC were pre-treated with a JAK1/Tyk2 inhibitor (18), STAT1 phosphorylation by IFNα was inhibited in a dose-dependent manner confirming its efficiency at inhibiting downstream IFNAR signaling (**Figure 4B**). We confirmed the expression of the ISG RSAD2 was inhibited by TS treatment (**Figure 4C**). Importantly, the inhibitory effect of IFNα on GILZ and DEX-induced GILZ was released by treatment with TS, demonstrating that JAK1/Tyk2 mediates inhibitory IFN signaling acting on GILZ expression. (**Figure 4D**).

**Figure 5:**
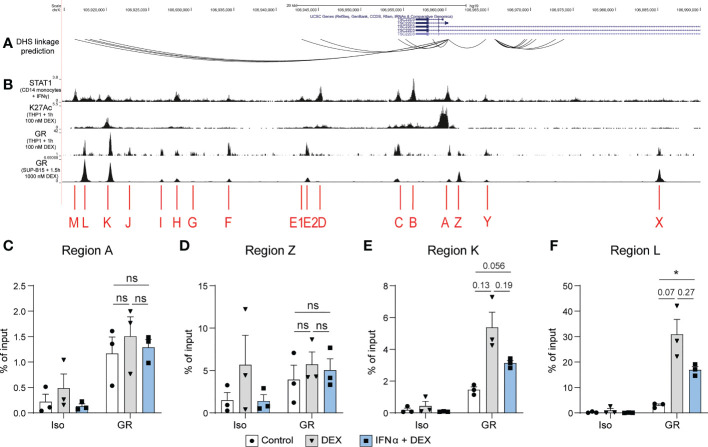
DEX-induced GR binding at a putative GILZ enhancer is reduced after IFNα pre-treatment. **(A)** DHS linkage predictions are mapped to indicate potential looping regulatory regions connected to the GILZ promoter (19). **(B)** ChIP-seq data of STAT1 binding in CD14+ monocytes treated with IFNγ (GSE99887), H3K27Ac histone acetylation and GR binding in THP1 cells treated with 100 nM DEX (GSE43036) and GR binding in SUP-B15 cells treated with 100 nM DEX (GSE107584). **(C–F)** ChIP for GR in L363 cells treated with DEX or IFNα and DEX. Enrichment was assessed in regions **(C)** A, **(D)** Z,**(E)** K and **(F)** L as indicated in figure B. n=3 from three independent experiments. ns, Not significant.

Finally, we examined the *TSC22D3* locus, the gene encoding GILZ protein, for potential transcription factor binding sites regulating its expression (**Figures 5A, B**). We identified DHS linkages (19) predicting physical looping of the chromatin between sections of the *TSC22D3* locus (**Figure 5A**). The presence of multiple loops indicated a putative enhancer region around 15kB downstream of the *TSC22D3* gene, which is confirmed by the presence of H3K27Ac peaks in this region (**Figure 5B**). Interestingly, by analyzing public datasets GSE99887, GSE107584 and GSE43036, we found multiple overlapping binding sites for STAT1 and GR in this potential enhancer region and in the TSC22D3 promoter region (**Figure 5B**). Therefore, we conducted ChIP in the human B cell line L363 to demonstrate GR binding at several of these sites in response to GC treatment and determine how this was affected by the presence of IFN. Regions A and Z, which are located around the promoter of *TSC22D3* isoform 1, showed minimal or no impact of IFN on GR binding (**Figures 5C, D**). However, Regions K and L, located in the novel enhancer region of the *TSC22D3* locus, showed a consistent but not significant reduction in Dex-induced GR binding when IFN was present (**Figures 5E, F**). This supports the concept that IFN directly interferes with GC-induced GILZ expression by activating STAT competition for GR binding sites at the GILZ locus.

## 4 Discussion

Glucocorticoids have formed a central part of SLE management for over seven decades. IFN has been shown to play a central role in SLE pathogenesis but GC are poor at suppressing the IFN pathway, and this is likely to play a role in GC resistance (8, 9). In this study we have shown that GILZ, an anti-inflammatory GC-induced protein, is suppressed by IFN. We provide evidence that downregulation of GILZ by IFN is JAK1/Tyk2 pathway dependent, and that the presence of IFN results in reduced GR binding in key regulatory regions of the *TSC22D3* gene, blunting GILZ induction by GC. Paired with the existing knowledge that GILZ regulates multiple other components of the immune system relevant to SLE pathogenesis, this work provides evidence that IFN regulation of GILZ may be a key mechanism involved in GC resistance in SLE.

Our work shows that IFN is associated with suppressed GILZ in multiple conditions, including *in vitro* studies of human PBMC, as well as *in vivo* in SLE patients, in patients with MS taking therapeutic IFN, and in IFN-overexpressing mice. While ability for IFN to downregulate GILZ expression has not before been reported, our group has previously shown that patients with active SLE have reduced GILZ expression in B lymphocytes and plasmablasts compared with healthy controls (12). Given the known association of active SLE with high IFN (20), this data is consistent with the concept that IFN downregulates GILZ in SLE.

Previous studies have alluded to a possible role for IFN in causing GC resistance. Patients with SLE have a blunted response to GC, as measured by a reduction in cortisol suppression post GC treatment compared to control subjects (21). A similar phenomenon has also been reported in patients receiving therapeutic IFNα treatment for hepatitis C (22). Clinically, patients with SLE often require high doses of GC to control their disease compared to patients with autoimmune conditions such as rheumatoid arthritis, in which IFN does not play a central role. Here we have shown that IFN suppresses GILZ induction by GC, and that the presence of IFN leads to reduced GR binding in regulatory regions of the *TSC22D3* locus, providing a mechanism *via* which IFN impairs a key GC immunoregulatory function. GILZ has been shown to mediate many of the anti-inflammatory effects of GC *via* inhibition of the key pro-inflammatory transcription factor NF-κB (23), as well as interactions with other mediators of inflammation such as transcription factor AP-1 (24) and inhibition of extracellular signal-related kinase (ERK) and PI3K-Akt signaling pathways (25, 26). GILZ has also been reported to have several cellular effects that are relevant to SLE pathogenesis, including a crucial role in B lymphocyte regulation (12) and effects on arthritis and leukocyte recruitment (27, 28). A predominance of Th17 cells and deficient or defective T regulatory cells have been reported in SLE (29, 30), and GILZ has been shown to both regulate the proliferation of Th17 cells (31), and favor T regulatory cell differentiation (32). Given GILZ has such key roles in mediating GC related anti-inflammatory effects, the reduced ability for GC to induce GILZ in high IFN states is likely to contribute to GC resistance in SLE.

Our findings suggest that the inhibition of GILZ by IFN occurs *via* the Jak/STAT pathway, as inhibition of this pathway ameliorated the effect of IFN on GC induction of GILZ. We also showed that inhibition of Tyk1/Jak2 with tofacitinib in lupus-prone mice produced an increase in GILZ expression, suggesting this pathway has an inhibitory effect on GILZ transcription. Consistent with this, anifrolumab, a therapeutic agent which inhibits the IFNAR receptor, and subsequently prevents the activation of Tyk1 and Jak2, was recently shown to have significant steroid-sparing effects in patients with active SLE in a successful phase 3 clinical trial (5). It will be valuable to assess potential restoration of GILZ expression by anifrolumab.

Previous studies have reported that the GR can directly interact with STAT proteins (33, 34). In our study, IFN seems to reduce binding of the GR at potential regulatory regions of the *TSC22D3* locus, which indicates that the GC/GR transcription factor and transcription factors downstream of Tyk1/Jak2, such as the STAT proteins or the ISGF-3 complex, are competitively binding at the GILZ locus or directly interact with each other to regulate GILZ transcription. Further work, including determining the functional role of the potential regulatory regions, is needed to delineate the mechanisms at play.

In conclusion, our work shows that in the presence of IFN, GC have reduced ability to induce expression of the key anti-inflammatory protein GILZ, and that blocking the function of IFN by targeting the JAK-STAT pathway restores GILZ induction by GC. GC resistance in SLE is problematic as higher doses of GC are required to control disease activity but cause detrimental metabolic effects. This work provides a mechanism by which IFN may induce GC resistance in SLE and highlights pathways which may be targeted in the development of steroid-sparing agents.

## Data availability statement

The original contributions presented in the study are included in the article/supplementary material. Further inquiries can be directed to the corresponding author.

## Ethics statement

The studies involving human participants were reviewed and approved by Monash Health HREC. The patients/participants provided their written informed consent to participate in this study. The animal study was reviewed and approved by Juntendo University Animal Ethics Committee.

## Author contributions

WD, MN, PH, EM, and SJ contributed to conception and design of the study. WD, MN, TB, AD’C, RS, LG, BR, IM, and SS conducted experiments and analyzed data. MF, KH, and KI provided data from experiments. WD and MN wrote the manuscript. WD, MN, EM, and SJ revised the manuscript. All authors contributed to the article and approved the submitted version.

## Acknowledgments

Work included in this manuscript was supported by grants from the Lupus Research Alliance (EM) and National Health and Medical Research Council of Australia (EM, MN, and SJ). Some data included in this manuscript were presented at the American College of Rheumatology congress, 2020 (abstract #0304).

## Conflict of interest

The authors declare that the research was conducted in the absence of any commercial or financial relationships that could be construed as a potential conflict of interest.

## Publisher’s note

All claims expressed in this article are solely those of the authors and do not necessarily represent those of their affiliated organizations, or those of the publisher, the editors and the reviewers. Any product that may be evaluated in this article, or claim that may be made by its manufacturer, is not guaranteed or endorsed by the publisher.

## References

[1] Petri M Orbai A-M Alarcón GS Gordon C Merrill JT Fortin PR. Derivation and validation of systemic lupus international collaborating clinics classification criteria for systemic lupus erythematosus. Arthritis rheumatism. (2012) 64(8):2677–86. doi: 10.1002/art.34473 PMC3409311 22553077

[2] Apostolopoulos D Kandane-Rathnayake R Louthrenoo W Luo S Wu Y-J Lateef A. Factors associated with damage accrual in patients with systemic lupus erythematosus with no clinical or serological disease activity: a multicentre cohort study. Lancet Rheumatol (2020) 2(1):e24–30. doi: 10.1016/S2665-9913(19)30105-5 38258272

[3] Bennett L Palucka AK Arce E Cantrell V Borvak J Banchereau J. Interferon and granulopoiesis signatures in systemic lupus erythematosus blood. J Exp Med (2003) 197(6):711–23. doi: 10.1084/jem.20021553 PMC2193846 12642603

[4] Psarras A Emery P Vital EM. Type I interferon-mediated autoimmune diseases: pathogenesis, diagnosis and targeted therapy. Rheumatol (Oxford England). (2017) 56(10):1662–75. doi: 10.1093/rheumatology/kew431 28122959

[5] Morand EF Furie R Tanaka Y Bruce IN Askanase AD Richez C. Trial of anifrolumab in active systemic lupus erythematosus. New Engl J Med (2020) 382(3):211–21. doi: 10.1056/NEJMoa1912196 31851795

[6] Barrat FJ Coffman RL. Development of TLR inhibitors for the treatment of autoimmune diseases. Immunol Rev (2008) 223:271–83. doi: 10.1111/j.1600-065X.2008.00630.x 18613842

[7] Ronnblom L Leonard D. Interferon pathway in SLE: one key to unlocking the mystery of the disease. Lupus Sci Med (2019) 6(1):e000270. doi: 10.1136/lupus-2018-000270 31497305 PMC6703304

[8] Guiducci C Gong M Xu Z Gill M Chaussabel D Meeker T. TLR recognition of self nucleic acids hampers glucocorticoid activity in lupus. Nature (2010) 465(7300):937–41. doi: 10.1038/nature09102 PMC2964153 20559388

[9] Northcott M Gearing L Nim H Nataraja C Hertzog PJ Jones SA. Glucocorticoid gene signatures in systemic lupus erythematosus and the effects of type I interferon: a cross-sectional and in-vitro study. Lancet Rheumatol (2021) 3(5):e357–370. doi: 10.1016/S2665-9913(21)00006-0 38279391

[10] Ayroldi E Macchiarulo A Riccardi C. Targeting glucocorticoid side effects: selective glucocorticoid receptor modulator or glucocorticoid-induced leucine zipper? a perspective. FASEB journal: Off Publ Fed Am Societies Exp Biol (2014) 28(12):5055–70. doi: 10.1096/fj.14-254755 25205742

[11] D’Adamio F Zollo O Moraca R Ayroldi E Bruscoli S Bartoli A. A new dexamethasone-induced gene of the leucine zipper family protects T lymphocytes from TCR/CD3-activated cell death. Immunity. (1997) 7(6):803–12. doi: 10.1016/S1074-7613(00)80398-2 9430225

[12] Jones SA Toh AE Odobasic D Oudin MA Cheng Q Lee JP. Glucocorticoid-induced leucine zipper (GILZ) inhibits b cell activation in systemic lupus erythematosus. Ann Rheum Dis (2016) 75(4):739–47. doi: 10.1136/annrheumdis-2015-207744 26612340

[13] Nataraja C Dankers W Flynn J Lee JPW Zhu W Vincent FB. GILZ regulates the expression of pro-inflammatory cytokines and protects against end-organ damage in a model of lupus. Front Immunol (2021) 12:652800. doi: 10.3389/fimmu.2021.652800 33889157 PMC8056982

[14] Livak KJ Schmittgen TD. Analysis of relative gene expression data using real-time quantitative PCR and the 2[-delta delta C(T)] method. Methods. (2001) 25(4):402–8. doi: 10.1006/meth.2001.1262 11846609

[15] Hoffman RW Merrill JT Alarcon-Riquelme MM Petri M Dow ER Nantz E. Gene expression and pharmacodynamic changes in 1,760 systemic lupus erythematosus patients from two phase III trials of BAFF blockade with tabalumab. Arthritis Rheumatol (2017) 69(3):643–54. doi: 10.1002/art.39950 PMC6585752 27723281

[16] Furie R Khamashta M Merrill JT Werth VP Kalunian K Brohawn P. Anifrolumab, an anti-interferon-alpha receptor monoclonal antibody, in moderate-to-Severe systemic lupus erythematosus. Arthritis Rheumatol (Hoboken NJ) (2017) 69(2):376–86. doi: 10.1002/art.39962 PMC5299497 28130918

[17] Ikeda K Hayakawa K Fujishiro M Kawasaki M Hirai T Tsushima H. JAK inhibitor has the amelioration effect in lupus-prone mice: The involvement of IFN signature gene downregulation. BMC Immunol (2017) 18(1):41. doi: 10.1186/s12865-017-0225-9 28830352 PMC5568047

[18] Fensome A Ambler CM Arnold E Banker ME Brown MF Chrencik J. Dual inhibition of TYK2 and JAK1 for the treatment of autoimmune diseases: Discovery of ((S)-2,2-Difluorocyclopropyl)((1 R,5 s)-3-(2-((1-methyl-1 h-pyrazol-4-yl)amino)pyrimidin-4-yl)-3,8-diazabicyclo[3.2.1]octan-8-yl)methanone (PF-06700841). J Med Chem (2018) 61(19):8597–612. doi: 10.1021/acs.jmedchem.8b00917 30113844

[19] Thurman RE Rynes E Humbert R Vierstra J Maurano MT Haugen E. The accessible chromatin landscape of the human genome. Nature (2012) 489(7414):75–82. doi: 10.1038/nature11232 22955617 PMC3721348

[20] Northcott M Jones S Koelmeyer R Bonin J Vincent F Kandane-Rathnayake R. Type 1 interferon status in systemic lupus erythematosus: a longitudinal analysis. Lupus Sci Med (2022) 9(1):e000625. doi: 10.1136/lupus-2021-000625 35197305 PMC8867321

[21] Melo AK Melo MR Saramago AB Demartino G Souza BD Longui CA. Persistent glucocorticoid resistance in systemic lupus erythematosus patients during clinical remission. Genet Mol research: GMR. (2013) 12(2):2010–9. doi: 10.4238/2013.February.19.1 23479142

[22] Felger JC Haroon E Woolwine BJ Raison CL Miller AH. Interferon-alpha-induced inflammation is associated with reduced glucocorticoid negative feedback sensitivity and depression in patients with hepatitis c virus. Physiol behavior. (2016) 166:14–21. doi: 10.1016/j.physbeh.2015.12.013 PMC4912474 26703235

[23] Ayroldi E GM Bruscoli S Marchetti C Zollo O Cannarile L Riccardi C. Modulation of T-cell activation by the glucocorticoid-induced leucine zipper factor *via* inhibition of nuclear factor kappaB. Blood. (2001) 98(3):743–53. doi: 10.1182/blood.V98.3.743 11468175

[24] Mittelstadt PR Ashwell JD. Inhibition of AP-1 by the glucocorticoid-inducible protein GILZ. J Biol Chem (2001) 276(31):29603–10. doi: 10.1074/jbc.M101522200 11397794

[25] Ayroldi E Zollo O Macchiarulo A Di Marco B Marchetti C Riccardi C. Glucocorticoid-induced leucine zipper inhibits the raf-extracellular signal-regulated kinase pathway by binding to raf-1. Mol Cell Biol (2002) 22(22):7929–41. doi: 10.1128/MCB.22.22.7929-7941.2002 PMC134721 12391160

[26] Ayroldi E Zollo O Bastianelli A Marchetti C Agostini M Di Virgilio R. GILZ mediates the antiproliferative activity of glucocorticoids by negative regulation of ras signaling. J Clin Invest. (2007) 117(6):1605–15. doi: 10.1172/JCI30724 PMC1865030 17492054

[27] Ngo D Beaulieu E Gu R Leaney A Santos L Fan H. Divergent effects of endogenous and exogenous GILZ in models of inflammation and arthritis. Arthritis Rheumatism. (2013) 65(5):1203–12. doi: 10.1002/art.37858 23335223

[28] Cheng Q Fan H Ngo D Beaulieu E Leung P Lo CY. GILZ overexpression inhibits endothelial cell adhesive function through regulation ofNF-κB and MAPK activity. J Immunol (2013) 31. doi: 10.4049/jimmunol.1202662 23729444

[29] Crispín JC Oukka M Bayliss G Cohen RA Van Beek CA Stillman IE. Expanded double negative T cells in patients with systemic lupus erythematosus produce IL-17 and infiltrate the kidneys. J Immunol (Baltimore Md: 1950). (2008) 181(12):8761–6. doi: 10.4049/jimmunol.181.12.8761 PMC2596652 19050297

[30] Chavele KM Ehrenstein MR. Regulatory T-cells in systemic lupus erythematosus and rheumatoid arthritis. FEBS letters. (2011) 585(23):3603–10. doi: 10.1016/j.febslet.2011.07.043 21827750

[31] Jones SA Perera DN Fan H Russ BE Harris J Morand EF. GILZ regulates Th17 responses and restrains IL-17-mediated skin inflammation. J Autoimmun (2015) 61:73–80. doi: 10.1016/j.jaut.2015.05.010 26077873

[32] Yang N Baban B Isales CM Shi XM. Crosstalk between bone marrow-derived mesenchymal stem cells and regulatory T cells through a glucocorticoid-induced leucine zipper/developmental endothelial locus-1-dependent mechanism. FASEB journal: Off Publ Fed Am Societies Exp Biol (2015) 29(9):3954–63. doi: 10.1096/fj.15-273664 PMC4550369 26038125

[33] Stöcklin E Wissler M Gouilleux F Groner B. Functional interactions between Stat5 and the glucocorticoid receptor. Nature (1996) 383(6602):726–8. doi: 10.1038/383726a0 8878484

[34] Hu F Pace TWW Miller AH. Interferon-alpha inhibits glucocorticoid receptor-mediated gene transcription *via* STAT5 activation in mouse HT22 cells. Brain behavior Immun (2009) 23(4):455–63. doi: 10.1016/j.bbi.2009.01.001 PMC2666112 19167480

